# Attitudes and Beliefs of the Italian Population towards COVID-19 Vaccinations

**DOI:** 10.3390/ijerph19106139

**Published:** 2022-05-18

**Authors:** Nadia Rania, Ilaria Coppola, Marta Brucci, Francesca Lagomarsino

**Affiliations:** Department of Education Sciences, University of Genoa, 16128 Genoa, Italy; ilaria.coppola@edu.unige.it (I.C.); marta.brucci@yahoo.com (M.B.); f.lagomarsino@unige.it (F.L.)

**Keywords:** attitudes and beliefs, COVID-19 vaccination, Italy, quantitative research

## Abstract

Background: Despite the numerous campaigns to encourage vaccination against COVID-19, the public debate and often conflicting information have left many individuals uncertain about the decision to make on whether or not to vaccinate. Methods: This research aims to analyze the attitudes and beliefs of the Italian population towards COVID-19 and other vaccinations through a quantitative methodology. In all, 500 adults (Age M = 39.52) participated in this exploratory study with an online questionnaire conducted in April 2021. Results: most participants believe vaccination is necessary to defeat COVID-19; there is an age-related difference in getting vaccinations, and women were more afraid of unexpected future effects than men; older participants have expressed a greater willingness to pay to be vaccinated against COVID-19 (4). Conclusion: In light of these results, it is necessary to pay greater attention to the perplexity and fears expressed by the population, especially women and youth, in relation to vaccinations; in fact, it would help to achieve a wider adherence to the tools designed to contain the spread of viruses at the base of severe health crises.

## 1. Introduction

One of the most controversial aspects of the pandemic is related to vaccination and the debate that has emerged around this topic has divided populations between those in favor and those opposed to receiving the vaccine [1]. The debate has left many individuals uncertain over how to proceed despite the knowledge that vaccinations are universally recognized as one of the most effective preventive measures for public health, as reported by Di Martino et al. [2]. Immunization programs make it possible to reduce preventable infectious diseases by decreasing morbidity, mortality and health costs [3,4].

Despite recent campaigns encouraging vaccination, the growing hesitation and outright rejection of other vaccines are now among the top ten threats to global health [5,6]. Within this framework, some authors highlight how herd immunity can only be achieved through high vaccination coverage among the population [7,8]. COVID-19 vaccines have been developed much faster than other vaccines in the past and this has caused widespread fears about their quality, efficacy and possible side effects, both immediate and long-term. In this regard, some authors [9,10,11,12] underline how the hesitation in accepting the COVID-19 vaccine is mainly due to the concerns on its safety and effects. Indeed, some research highlights possible side effects of the vaccine, such as myocarditis [13,14] and thrombosis [15]. Sessa [16] stated that it is necessary to study thoroughly these outcomes because they could be a “possible” consequence of COVID-19 vaccine. The research conducted by Napolitano et al. [17] highlighted that, among those who have a parental role, those who fear more about the safety and side effects of the vaccine are the ones who hesitate more in having their children vaccinated. However a lot of research finds COVID-19 vaccination to be safe with only minor adverse effects [18,19] even among patients with particular diseases [20], pregnant women [21] and children [22].

According to Sabahelzain et al. [23], the willingness to accept COVID-19 vaccines is dynamic and depends on the perceived risk and the immediate threat when deciding in favor or against the vaccine. Furthermore, the population is also influenced by the positions that virologists and politicians take when discussing the pandemic on social media. Although vaccination is known to offer immunity to the population, resulting in the resumption of routine social and economic activities [24,25], if it is not widely accepted by the population, it is ineffective [26]. As reported by Schoch-Spana et al. [25], even in previous crisis situations, for various reasons, vaccines were not accepted by the entire population as necessary measures to combat the spread of diseases [27,28]. 

## 2. Italian Context

In Italy, the phenomenon of low national vaccination rates, which have been decreasing since 2013, was addressed with the introduction of law no. 119/2017 which provides for mandatory vaccinations for ten infectious diseases [29]. Within this scenario, the vaccination campaign for COVID-19 has moved to protect public health. The National Strategic Plan for anti-SARS-CoV-2/COVID-19 vaccination was adopted by Ministerial Decree on 2 January 2021 and was based on the constitutional provision that refers to the principles of equity, reciprocity, legitimacy, protection, well-being and promotion of health. During the first phase, because the supply of vaccines was limited, some priorities were put in place to reduce mortality and morbidity [30].

Thus, the decision to accept the new COVID-19 vaccines is a complex situation that brings into play resistance and fears related to more widely used and tested vaccines for other diseases. Furthermore, as stated by Cadeddu et al. [31], studies on the opinions of Italians regarding beliefs on vaccinations are rare, especially those regarding the COVID-19 pandemic. Starting from this theoretical framework, the choice to carry out this research stems from the fact that, at the time of the data collection, the Comirnaty vaccine (Pfizer) was reserved for healthcare professionals, elderly and fragile patients, while AstraZeneca (later renamed Vaxzervria) was designated for others. However, the first cases of thrombosis following the administration of AstraZeneca were reported in the media with alarm, and the use of the vaccine was suspended by the European Medicines Agency (EMA), raising concerns about the safety of the entire campaign.

## 3. Aims

Starting from this theoretical framework, the aim of the research was to understand who among the general population would get vaccinated if given the opportunity. Another aim was to investigate participants’ attitudes, beliefs and level of understanding of information regarding the vaccine for COVID-19 and other vaccines. Moreover, we wanted to analyze the differences in relation to sociodemographic variables (sex, age, educational qualification, marital status and current working methods) and correlations between the dimensions studied.

## 4. Materials and Methods

### 4.1. Procedure

The present research follows a multidisciplinary approach; it is based on a quantitative and exploratory theoretical framework to give a picture of the perceptions of the population in relation to their attitudes and beliefs toward general and COVID-19 vaccination.

The data was collected for one month, in April 2021; approximately two-thirds of the questionnaires were compiled on the first three days of the questionnaire launch. Only fully completed questionnaires were analyzed. The study questionnaire was administered online. The platform used for the questionnaire was Microsoft Forms. The questionnaire was previously filled in by the researchers via links to verify its functionality and feasibility on smartphone and computer. Both mobile phone and computer displayed 4 questions per page for a total of 6 pages. For each page there was the possibility to go back to verify or modify the answers given. Participants were able to complete the survey in about 20 min. Participants were invited to fill out the questionnaire via a link sent by email and in posts on WhatsApp, discussion forums and social networks such as Facebook. The inclusion criteria were being at least 18 years old, living in Italy and speaking fluent Italian (language used for the creation of the questionnaire). The convenience sample was recruited through random cascade sampling, starting from subjects known by the research team. The questionnaire was proposed throughout Italy, thanks to its dissemination using social media; however, most of the participants who completed the questionnaire were from the same region as those who conducted the research.

Participants joined on a voluntary basis, without any type of incentive. Before completing the questionnaire, on an introductory page, the objectives of the study and the proposed topics were shared. The participants were asked to read and accept an informed consent, through which it was made it clear that they had the possibility of withdrawing from participation at any time by closing the browser window and that the data would be processed exclusively in aggregate form. They were informed that the GDPR (EU Regulation no. 2016/679) provides for the protection of persons regarding the processing of personal data. Only by accepting the consent could the participants access the questionnaire. Additionally, each participant was asked to build a code so they could be contacted for further research. The code, therefore, made it possible to verify that the same participant did not fill in the proposed questionnaire several times.

The data was collected and stored in accordance with the ethical recommendations of the Declaration of Helsinki and in compliance with the American Psychological Association (APA) standards for the treatment of human volunteers. This research represents a second survey titled “1 year of COVID-19: how we are and how we deal with it”. There was, in fact, a first survey titled “COVID Emergency-19-How I feel and how I live it”, where the psychological consequences of COVID-19 were investigated, and for which we had obtained the approval of the ethics committee of the Department of Education Sciences (Approval code: 039 Year of Approval: 2020). The second survey, object of this article, is an integral part of the first research as it includes the protocol present in the first survey, to which some validated scales in the literature relating to attitudes and beliefs towards vaccines have been integrated.

The research, of an exploratory nature, does not want to return a representative image of the Italian population, but rather highlight the attitude and beliefs of the population towards vaccination against COVID-19 and other vaccinations.

### 4.2. Measures

The online protocol had four main sections: demographic characteristics; attitudes and beliefs toward general vaccination; skills, perceptions, attitudes and beliefs toward COVID-19.

The demographic section was composed of seven items exploring the characteristics of participants, their education level, and information about their work during the COVID-19 pandemic.

The section on the attitudes and beliefs toward general vaccination included twelve items from Di Martino et al. [2]; the answers were scored on a Likert scale, where 4 to 5 points were considered “agreement” with the item, whereas scores between 1 and 3 were considered “disagreement”). The next part of the questionnaire is based on the research of Biasio et al. [32]: there are 3 items that evaluate current vaccine behavior (Current vaccine behavior) (categorical yes/no) and 2 items that evaluate beliefs about vaccination (Beliefs about vaccination), all of which are scored on a 4-point Likert scale.

The third section of the survey comprised tools employed to assess skills, perceptions, attitudes, and beliefs toward COVID-19 vaccinations; this part is composed by 17 items of Biasio et al. [32], and includes Vaccine literacy (VL) with 4 items to evaluate functional skills (VL functional skills) (α = 0.86) and 8 items to evaluate interactive/critical skills (VL interactive/critical skills) (α = 0.77), with all items scored on a 4-point Likert scale. VL functional skills questions were mainly about language, involving the semantic system, while the VL interactive/critical skills questions focused more on cognitive efforts, such as problem-solving and decision-making, with higher scores corresponding to higher VL levels. An additional 5 items of Biasio et al. [32] evaluated COVID-19 vaccines perceptions and attitudes (COVID-19 vaccines perceptions and attitudes) (categorical yes/no). These items were adapted, as vaccines had already been created by the time the questionnaire was administered. This section of the survey also employs the Vaccination Attitudes Examination Scale [6], which includes 12 items scored on a 6-point Likert scale (from strongly disagree to strongly agree) and assessing vaccination attitudes specific to COVID-19. These questions are divided into four subscales: Mistrust of vaccine benefit (VA_M) (α = 0.94); Worry about unforeseen future effects of vaccine (VA_F) (α = 0.9); Concerns about commercial profiteering (VA_CP) (α = 0.7); Preference for natural immunity (VA_NI) (α = 0.85). A further question probed whether participants had received the vaccine, planned to receive the vaccine or did not intend to get the vaccine (COVID-19 vaccination/intentions). Table 1 shows a summary of the measures used.

### 4.3. Data Analysis

Descriptive statistics were calculated for sociodemographic characteristics and information about variables, consisting of frequencies and percentages. The scores of variables investigated (VL functional skills and VL interactive/critical skills, Mistrust of vaccine benefit, Worry about unforeseen future effects of vaccine, Concerns about commercial profiteering and Preference for natural immunity) were expressed as means and standard deviations. The dichotomic variable, gender, in relation to VL functional skills, VL interactive/critical skills and subscales Mistrust of vaccine benefit, Worry about unforeseen future effects of vaccine, Concerns about commercial profiteering and Preference for natural immunity were evaluated with *t*-tests for independent samples. Instead, in relation to Current vaccines behavior, COVID-19 vaccines perceptions and attitudes, Beliefs about vaccination and COVID-19 vaccination/intentions were evaluated using Chi-Square tests.

To compare the differences between our participants and the Italian normative sample for VL functional skills and VL interactive/critical skills scales, *t*-tests were conducted for single samples.

Chi-Square tests were used to examine differences between groups (age, marital status, current workplace arrangements and educational qualification) in regard to Current vaccine behavior, Beliefs about vaccination, COVID-19 vaccines perceptions and attitudes, and COVID-19 vaccination/intentions and analysis of variance in relation to VL functional skills, VL interactive/critical skills, and the categories Mistrust of vaccine benefit, Worry about unforeseen future effects of vaccine, Concerns about commercial profiteering and Preference for natural immunity. A post hoc Tukey (for homogeneous variances) or Games-Howell (for nonhomogeneous variances) test was administered for between-group comparisons in case of a significant overall F-value. Appropriate effect size statistics that adjusted for differences in group sizes were obtained with Hedges’g for *t*-tests and η^2^ for ANOVAs.

To explore the relationship between VL functional skills and VL interactive/critical skills and the Mistrust of vaccine benefit subscales, concerns about commercial profiteering and preference for natural immunity scales, correlation analyses (Pearson correlation coefficient r) were conducted. A multiple linear regression was calculated to predict Mistrust of vaccine benefit based on attitudes and beliefs toward general vaccination and skills, perceptions, attitudes, and beliefs toward COVID-19 vaccinations.

To determine statistical significance *p* value of 0.05 two-sides tests were used.

## 5. Results

### 5.1. Participants

A total of 500 adults from all over Italy participated in the online questionnaire. Most respondents were women (86%), young adults (age M = 39.52 years, SD = 16.58; range 20–89), single (47.7%) or married or cohabiting with partner (44.7%), without children (62.4%), with unchanged current workplace arrangements (67.9%) and secondary education levels (41.9%), followed by those who held at least a university degree (39%). Regarding the willingness to vaccinate, 85.5% of men had received the vaccine or were awaiting their turn, 10.1% were undecided whether to be vaccinated and 4.3% had not received the vaccine and did not intend to receive the shot. Regarding women, 81.6% had received the vaccine or were awaiting their turn, 13.7% were undecided whether to be vaccinated and 4.7% had not received the vaccine and did not intend to receive the shot. Furthermore, most participants who reported having children (35.3%) were in the 55–64 age group.

As for proximity to COVID-19, 12.3% of participants contracted it, while 29.4%, 50.5% and 40.6%, respectively, had a family member, friend or acquaintance who contracted COVID-19, but who survived; moreover, in lower percentages the participants reported having had a family member (5.8%), a friend (5.6%) or an acquaintance (27.2%), who contracted COVID-19 and who did not survive.

The post hoc analyses revealed the statistical power of this study was 0.67 for detecting a small effect, whereas the power exceeded 0.99 for the detection of a moderate to large effect size. Thus, there was more than adequate power (0.99) at the moderate to large effect size level, but less than adequate statistical power at the small effect size level [33]. In Table 2, we report the sociodemographic characteristics of the participants in detail.

### 5.2. Descriptive Statistics

The results illustrate the opinions of Italians related to attitudes and beliefs toward vaccination in general and more specifically, skills, perceptions, attitudes and beliefs toward COVID-19 vaccination.

Table 3 reports the mean and standard deviation for the analysis of skills, perceptions, attitudes and beliefs toward COVID-19 vaccinations; vaccine literacy (VL functional skills and VL interactive/critical skills); and subscales of the Vaccination Attitudes Examination Scale: Trust/Mistrust of vaccine benefit, Worries over unforeseen future effects, Concerns about commercial profiteering and Preference for natural immunity.

### 5.3. Sociodemographic Variables

#### 5.3.1. Gender

Regarding gender, the analysis of the results did not reveal significant differences regarding Current vaccine behavior, Beliefs about vaccination (attitudes and beliefs towards general vaccination), vaccine literacy, COVID-19 vaccine perceptions and attitudes and COVID-19 vaccination/intentions (skills, perceptions, attitudes and beliefs toward COVID-19 vaccinations).

Regarding attitudes and beliefs toward general vaccination, in relation to the attitude toward the item by Di Martino et al. [2], “I’m afraid of the side effects of vaccinations”, a significant difference emerged between men and women (χ^2^ (4, 492) = 16.05, *p* = 0.003): 36.88% of women agreed with this statement compared to 18.84% of men. When prompted, “I’m afraid of getting sick after being vaccinated”, there was a significant difference between men and women (χ^2^ (4, 492) = 16.23, *p* = 0.003), as 13.71% of women agreed with this statement compared to 5.8% of men. Finally, to confirm these results, we examined the differences between males and females regarding the skills, perceptions, attitudes, and beliefs toward COVID-19 vaccinations, particularly the Worries over unforeseen future effects (Vaccination Attitudes Examination Scale) subscale; females reported a higher score on average (M = 3.87, SD: 1.29) than males (M = 2.97, SD = 1.48), t(490) = −5.26, *p* < 0.001 in Table 4. Descriptive statistics of variables in the sample and gender differences in responses (N = 500).

As reported in Table 5, more respondents were willing to be vaccinated against COVID-19 than vaccination against influenza; however, women (81.6%) were less likely than men (85.5%) to have either vaccination. The gender difference is also maintained with regard to vaccination against influenza: 33.3% of men said they had vaccinated against 26.7% of women.

#### 5.3.2. Age

Considering the different age groups, no significant differences emerged in reference to the items of Di Martino et al. [2] and Beliefs about vaccination (attitudes and beliefs toward general vaccination), or with regard to Vaccine Literacy (skills, perceptions, attitudes and beliefs toward COVID-19 vaccinations).

In relation to attitudes and beliefs toward general vaccination, relative to the Current vaccine behavior, and, in particular, to the prompt, “You were vaccinated against the flu last season”, a significant difference emerged regarding age: participants over 65 years of age (75.6%) were the highest group among those vaccinated for influenza, while the 18–24 age group (12.3%) was the lowest, χ^2^ (5, 497) = 77.29, *p* < 0.001.

When asked “Do you expect to be vaccinated against other infectious diseases?”, a significant difference emerges: 71% of respondents over 65 answered yes as did 68.5% of those aged 18–24, compared to 60.8% of those aged 25–34, 50% of those aged 35–44, 47.6% of those aged 55–64 and 39.1% of those aged 45–54, χ^2^ (5, 497) = 23.86, *p* < 0.001.

Regarding the skills, perceptions, attitudes and beliefs toward COVID-19 vaccinations, respondents were asked, “Are the vaccines produced safe and effective?” a significant difference emerged in relation to age: 85% of those aged 25–34 answered yes compared to 84.4% of those aged 65 and over, 80% of those aged 18–24, 79.8% of those aged 55–64, 74.1% of those aged 35–44 and 64.1% of those aged 45–54, χ^2^ (5, 497) = 12.85, *p* = 0.025.

Likewise, the question, “Would you pay to be vaccinated?” elicited a significant difference among the age groups: 66.7% of those aged 65 and over answered yes, compared to 50% of those aged 55–64, 44.6% of those aged 18–24, 40.6% of those aged 45–54, 39.2% of those aged 25–34 and 35.2% of those aged 35–44, χ^2^ (5, 497) = 13.64, *p* = 0.018. The question “Should children also be vaccinated?” also showed a significant difference among age groups: 83.1% of those 18–24 years old and 80% of those aged 25–34 answered yes, compared to 75.6% and 73.8% of those over 65 and 55–64, respectively, and 53.7% of those aged 35–44 and 40.6% of those aged 45–54, χ^2^ (5, 497) = 51.64, *p* < 0.001.

Additionally, with reference to the Vaccination Attitudes Examination Scale, and in particular in the Preference for natural immunity subscale, a significant difference emerged between those in the 45–54 age group and the other age groups with the exception of the 35–44 age group: participants aged 45-54 scored higher (M = 2.70, SD = 1.24) than those aged 18–24 (M = 2.14, SD = 0.1), 25–34 (M = 2.06, SD = 1.0), 55–64 (M = 2.1, SD = 1.04) and 65 and above (M = 1.96, SD = 0.98) F (5, 491) = 4.1, *p* = 0.001, η^2^p = 0.04.

Regarding the intention to vaccinate against COVID-19, a significant difference emerged: older individuals are the most likely to get vaccinated or already be vaccinated (95.6%), followed by those aged 55–64 (94%). However, young people, particularly those aged 25–34 (83.3%) and 18–24 (76.2%) demonstrate a greater propensity for vaccination; the percentage drops between those aged 45–54 (75%) and those aged 35–44 (72.2%), χ^2^ (10, 497) = 26.52, *p* = 0.003. With regard to the flu vaccine, on the other hand, the rate increase linearly with age: 12.3% of those between 18 and 24 years of age declared they had been vaccinated, 19.2% of those between 25 and 34 years old, 27.8% of those aged 35–44, 25% between 45–54 years, 39.3% of those aged 55–64 and 75.6% of those aged 65 or over. Table 6 reports the comparison between vaccination for influenza and COVID-19 by age group.

#### 5.3.3. Marital Status

Analyzing the marital status variable, no significant differences emerged in relation to either the current vaccination and belief in vaccination (attitudes and beliefs toward general vaccination) or Vaccine Literacy, Vaccination Attitudes Examination Scale and COVID-19 vaccination/intentions (skills, perceptions, attitudes and beliefs toward COVID-19 vaccinations).

Regarding attitudes and beliefs toward general vaccination, a significant difference emerges in response to the item by Di Martino et al. [2], “I am wary of the long-term effects of vaccinations on health”. Almost a quarter (22.6%) of divorced people agree with this statement compared to 19% of married respondents, and 18.5% of celibates χ^2^ (12, 497) = 22.16, *p* = 0.036. About the skills, perceptions, attitudes, behavior and beliefs toward COVID-19 vaccinations, and regarding the COVID-Vax scale, the question “Should children also be vaccinated?” elicited significant differences regarding marital status: 78.5% of respondents who were unmarried answered yes, and the percentage gradually decreased from the married/cohabitant (66.7%) and widow (57.1%) groups to the separated/divorced (54.8%) group χ^2^ (3, 497) = 13.12, *p* = 0.004.

#### 5.3.4. Current Workplace Arrangements

From the analysis of the variable current working methods, no significant differences emerged for the constructs investigated, neither regarding the dimension of attitudes and beliefs toward general vaccination nor for the dimension of skills, perceptions, attitudes and beliefs toward COVID-19 vaccinations.

#### 5.3.5. Level of Education

Concerning current vaccine behavior, beliefs about vaccination, (attitudes and beliefs toward general vaccination), VL functional skills, COVID-19 vaccine perceptions and attitudes, vaccination attitude examination scale and COVID-19 vaccination/intentions (skills, perceptions, attitudes and beliefs toward COVID-19 vaccinations), no significant differences emerged in relation to educational qualification. Instead, a significant difference emerged in relation to the item by Di Martino et al. [2], “I believe that vaccinations among health care workers are a prerequisite for working in the health sector”. All respondents with a lower secondary school education agreed with this statement compared to 80.8% of those with a high school diploma, 82.5% of those with an undergraduate degree and 71.9% of those with a postgraduate degree χ^2^ (12, 497) = 22.84, *p* = 0.03.

With regard to the skills, perceptions, attitudes and beliefs toward COVID-19 vaccinations, in particular in the VL interactive/critical skills, a difference emerged between respondents with a high school diploma (M = 3.22, SD = 0.46) and those with a postgraduate degree (M = 3.45, SD = 0.51): the latter obtained a higher score than the former *p* = 0.003, F(3, 493) = 4.73, η2 = 0.03.

### 5.4. Correlations

As seen in Table 7, a positive correlation emerges in the skills, perceptions, attitudes and beliefs toward COVID-19 vaccinations between the subscales of the Vaccination Attitudes Examination Scale and between VL functional skills and the subscales Worries over unforeseen future effects, Concerns about commercial profiteering and Preference for natural immunity. Furthermore, a negative correlation emerges between VL interactive/critical skills and the Mistrust of vaccine benefit, Worries over unforeseen future effects, Concerns about commercial profiteering and Preference for natural immunity subscales.

### 5.5. Regression

The step-wise progressive regression analysis was used to investigate the role of attitudes and beliefs toward general vaccination and skills, perceptions, attitudes, and beliefs toward COVID-19 vaccinations on Mistrust of vaccine benefit. In particular, some aspects of attitudes and beliefs toward general vaccination (“I believe vaccines are important in reducing or eliminating serious diseases”, “I believe more in natural immunity acquired through disease than in vaccines” and “I’m afraid of getting sick after getting vaccinated”) and Concerns about commercial profiteering, VL interactive/critical skills were entered as predictors. The model of regression, reported in Table 8, predicted 31% of the variance. The structural parameters of the model with the intercept are highly significant (*p* < 0.001).

## 6. Discussion

The strength of this study is the adequate number of participants even if biased by gender; as often happens in research, especially online [34], women are more involved in the processes of knowledge and participation [35] and respond to surveys in greater numbers than men. Using validated tools, this study focused both on vaccination coverage and on attitudes and beliefs toward vaccines. The majority of participants agreed with the idea that vaccinations are a necessary tool to defeat COVID-19, in line with Falcone et al. [36] and Fisher et al. [37], and in contrast with results of Kabir et al. [38].

### 6.1. Gender

Concerning other vaccinations, it emerges from our results how men were more likely to be vaccinated, less afraid of the side effects and less fearful of getting sick after being vaccinated than women; also with regard to vaccination for COVID-19, women were most afraid of unexpected future effects, and all of these findings were in keeping with the literature [9,10,26,39]. Di Giuseppe et al. [40] also underlined that men who did not marry or cohabitate perceived a lower risk of coming down with COVID-19 and declared that they did not want more information about the vaccine. In this regard, Sprengholz et al. [41] found that men and older people are more in favor of mandatory vaccines than women and young people.

### 6.2. Age

Concerning age, it emerges that in relation to other vaccination, those who are ages 45–54 as a percentage have a preference for natural immunity compared to other age groups; moreover, contrary to the literature [7,26,39], the majority of young people 25–34 and people over age 65 have shown more confidence in the safety and efficacy of vaccines than those aged 45–54, which instead is the group that showed less confidence in the vaccine (in fact only 64% answered yes to the question about vaccine safety and efficacy) and preferred natural immunity. People over the age of 65 made up the largest percentage of groups that expressed a willingness to pay to be vaccinated against COVID-19, which is probably because they are the most at risk of serious consequences caused by COVID-19. In fact, as found in the literature [42], being elderly, the worry of contracting COVID-19 and considering vaccination important for one’s health and that of the community are determining factors for the acceptance of vaccination. Regarding vaccination for children, the group with the largest number of respondents in favor was the youngest, aged 18–34 years, while the most reluctant were those aged 45–54; this could be since it corresponds to one of the two age groups among the participants who most reported having children and therefore more than others could feel the safety of their offspring threatened. In fact, even in the literature [17,43] it is highlighted how parents are hesitant to vaccinate their children due to safety and possible side effects of vaccination.

The issue of vaccines for minors is an extremely complex issue [44]. Even more than for adults, the idea of vaccinating a child or being forced to vaccinate a child brings up fears and ideological beliefs that existed long before COVID-19, for example, in the case of mandatory vaccination for measles. Health policy issues, such as Law 119 of 2017 in Italy also made additional vaccines mandatory for children from 0 to 16 years of age. In this regard, Wang et al. [10] reported that the refusal and hesitation to accept the COVID-19 vaccine by women can be an obstacle to COVID-19 vaccination for children since women usually make the choice of whether to vaccinate children. A research conducted by Dubé et al. [43] also found that a large proportion of the participants in a maternal role were reluctant to vaccinate their children and many relied on information accessible on the internet to make decisions about it. Finally, older people receive the most influenza vaccinations and are the largest number of respondents who plan to vaccinate against other infectious diseases including COVID-19, which is in line with the findings of Wang et al. [10].

### 6.3. Marital Status

Regarding marital status, most people who think children should be vaccinated are single, while the percentage declines among married, widowed and separated respondents; this may be because those who have children are more concerned about their health; moreover, those who are divorced report being more wary of the long-term effects of vaccinations on health.

### 6.4. Current Workplace Arrangements and Level of Education

No significant differences emerge in relation to current workplace arrangements. Concerning differences on level of education, respondents with a postgraduate degree, on average, scored higher in VL interactive/critical skills than those with a high school diploma, thus demonstrating that they have greater problem-solving and decision-making skills. Instead, in relation to other vaccination, the degree of agreement with the statement “I believe that vaccinations among health care workers are a prerequisite for working in the health sector” decreases in percentage as level of education increased; as also highlighted by Wang et al. [10], resistance from health professionals could influence decision by the general population.

### 6.5. Correlation and Regression

Furthermore, in relation to COVID-19 vaccination, a positive correlation emerged between VL functional skills and the subscales: Worries over unforeseen future effects, Concerns about commercial profiteering, Preference for natural immunity; a negative correlation emerged between VL interactive/critical skills and the subscales: Mistrust of vaccine benefit subscales, Worries over unforeseen future effects, Concerns about commercial profiteering and Preference for natural immunity. Biasio et al. [32] suggested that the amount of information available has stimulated many individuals to seek accurate and reliable information, comparing themselves with others and verifying the credibility of sources, thus increasing their critical ability and interactivity.

Finally, from regression analysis it emerges how the belief that vaccines are important for reducing or eliminating serious disease, commercial profit, the belief in the efficacy of natural immunity acquired through disease, fear of getting sick after getting vaccinated, and VLICS are factors that influence Mistrust of vaccine benefit. One factor affecting the level of confidence in COVID-19 vaccines is the level of accessibility and understanding of information. In regard to being vaccinated, Aksu and Öztürk [45] highlight how discussions on television or on social media could be a source of stress in relation to getting vaccinated and this can generate mistrust toward COVID-19 vaccination. Furthermore, our data, collected when the vaccine for COVID-19 had already been created, confirms the resistance that emerged from a research carried out before the creation of the vaccine for COVID-19, which revealed a correlation between vaccine rejection and mistrust of vaccine benefit [6]. From research conducted by Paul et al. [9] it emerges that the concerns related to the safety of the vaccine are also due to the lack of trust placed in the government. The authors, therefore, believe it essential to establish other communication channels managed by leaders recognized by the community.

### 6.6. Limitation and Strengths of the Research

There are several limitations that emerged from our work. The main limitation was the online questionnaire; although in this period of prolonged health crisis, virtual contact was one of the main methods of data collection, as highlighted in the literature [46,47,48], bias can result from inattentive response. Furthermore, in relation to the study design, a limit that hinders the generalization of the results is represented by the average age of the participants: in fact, the administration of the online questionnaire may have excluded that part of the population not inclined to use the technology. Another limit is the random cascade sampling that may not have allowed the involvement of some target populations not easily reached with online surveys, for example, the immigrant population or people less inclined to use technology. This being a research on a very current topic, it cannot be excluded that there are biases due to social desirability. Moreover, the research, being of an exploratory nature, does not return a representative picture of the Italian population, but aims to provide a picture of the perceptions of the population during the pandemic emergency in relation to the attitude and conviction towards vaccination against COVID-19. Another limit is due to the fact that a pilot study was not conducted before; even if most of the questions were related to the present, some items asked the participant to refer to a recent past and this may have caused a recall bias. Although interesting results emerge in relation to gender difference, it should be emphasized that most of the participants were female, as already highlighted in other researches [33,34]. This over-representation could be due to the fact that women, as shown by the data, are more concerned about the side effects of vaccines; therefore, they may have felt more involved in this type of research. A further limitation could be due to the self-selection bias; in fact, the participants were not chosen, but it was they who chose to join the research.

In spite of these weaknesses, this work has its strengths, because it was one of the first studies conducted in the general Italian population on vaccines with a multidisciplinary approach. Although a considerable amount of time has elapsed since data collection, the results may be useful to highlight the need to pay more attention to the causes and factors influencing the distrust of vaccines, the understanding of which is essential to obtain a wider acceptance of the population in the measures put in place to counter any future health crises.

## 7. Conclusions

The historic pandemic we are facing has impacted global health, economies and societies. However, from an analysis of attitudes and beliefs towards the tools used to contain the pandemic and, in particular, towards vaccines, it emerged that people are demonstrating greater awareness and confidence in the tools of medicine regardless of gender, age and the socio-economic status to which they belong. Concerns remain linked to the long-term effects that a still poorly understood vaccine could bring. The rapid time to test and implement the vaccine has created some concern in many people about the reliability and safety of the vaccine. This is a fact that should give pause to the need to communicate and share more clearly the information about vaccinations, needed to gain wider acceptance, even from those who show more fear and perplexity about side effects. Finally, another fundamental fact concerns the interest on the part of the participants in the search for information on vaccines, the concerns about the possible economic profits deriving from them and the fear of side effects, which seem to influence the level of distrust that one has towards the vaccines benefits of vaccination. It is therefore evident that institutions must pay more attention in communicating information relating to the need to be vaccinated, as the population has more and more information channels available through which they structure their beliefs. It seems evident that the clarity of the information shared by the Institutions and the availability of reliable and accessible resources could be a key variable in the voluntary use of protections, such as the vaccine.

## Figures and Tables

**Table 1:** The measure of the study.

Section		Sample Questions/Items	Author(s)	Assessment Score
Demographic characteristics	7 items Gender, Age, Marital status Children, Level of education Current workplace arrangements unchanged Proximity to COVID-19			
Attitudes and beliefs toward general vaccination	12 items (Attitudes and beliefs toward general vaccination)	*I believe vaccines are important for reducing or eliminating serious diseases*	DiMartino et al. [2]	Numerical 4-point Likert scale
	3 items (Current vaccine behavior)	*Have you been vaccinated against flu last season?*	Biasio et al. [32]	categorical yes/no
	2 items (Beliefs about vaccination).	*I am not favorable to vaccines because they are unsafe*		Numerical 4-point Likert scale
Skills, perceptions, attitudes, and beliefs toward COVID-19	17 items: 4 items VL functional skills 8 items VL interactive/critical skills	*When reading or listening to information about future COVID-19 vaccines or current vaccines, Did you find words you didn’t know?*		Numerical 4-point Likert scale
		*When looking for information about future COVID-19 vaccines or current vaccines, have you consulted more than one source of information?*		
	5 items COVID-19 vaccines perceptions and attitudes	*Regarding the COVID-19 vaccine, are the vaccines produced safe and effective?*		categorical yes/no
	12 items (Vaccination Attitudes Examination Scale) Mistrust of vaccine benefit (VA_M) Worry about unforeseen future effects of vaccine (VA_F) Concerns about commercial profiteering (VA_CP) Preference for natural immunity (VA_NI)	*I would feel safe once I got vaccinated against the virus causing COVID-19* (reversed)	Taylor et al. [6]	Numerical 6-point Likert scale
		*I would worry that there could be problems with a COVID-19 vaccine that have not yet been discovered*		
		*Authorities would promote COVID-19 vaccination for financial gain, not for people’s health*		
		*Natural exposure to the virus causing COVID-19 gives the safest protection*		
COVID-19 vaccination/intentions	1 item COVID-19 vaccination/intentions	*We ask you to indicate whether you have already received the vaccine, or if you intend to have the vaccine or not*		Categorical

**Table 2:** Sociodemographic characteristics of the participants (N = 500).

Category Variables	%
*Gender*	
male	14
female	86
*Age*	
18–24	26.2
25–34	24.1
35–44	10.9
45–54	12.9
55–64	16.9
65 or older	9
*Marital status*	
unmarried	47.7
married/cohabiting	44.7
separate/divorced	6.2
widower	1.4
*Children*	
People who have children	37.6
People who have no children	62.4
*People by age group who have children*	
18–24	0
25–34	4.3
35–44	13.9
45–54	26.2
55–64	35.3
65 and older	20.3
*Level of Education*	
junior high school	1.2
secondary school	41.9
graduation	39
postgraduate specialization	17.9
*Current workplace arrangements*	
unchanged	67.9
smart-working	26.4
loss of job/work permit/leave	5.7
*Proximity to COVID-19*	
COVID-19—firsthand experience	12.3
family members (have had COVID-19) and are healed	29.4
friends (have had COVID-19) and are healed	50.5
acquaintances (have had COVID-19) and are healed	40.6
family members (have had COVID-19) and did not survive	5.8
friends (have had COVID-19) and did not survive	5.6
acquaintances (have had COVID-19) and did not survive	27.2

**Table 3:** Mean and standard deviation for the analysis of skills, perceptions, attitudes and beliefs toward COVID-19 vaccinations.

Scales	M	SD
VL functional skills	2.21	0.71
VL interactive/critical skills	3.3	0.48
Trust/mistrust of vaccine benefit	2.9	1.33
Worries over unforeseen future effects	3.73	1.36
Concerns about commercial profiteering	2.93	1.16
Preference for natural immunity	2.18	1.06

**Table 4:** Descriptive statistics of variables and gender differences.

Socio Demographic Variable	Functional VL		Interactive Critical VL		Worries over Unforeseen Future Effects					Mistrust of Vaccine Benefit		Concerns about Commercial Profiteering		Preference for Natural Immunity	
	M	SD	M	SD	M	SD	t	p	Hedges’g	M	SD	M	SD	M	SD
*Gender*															
Male	2.18	0.70	3.22	0.54	2.97	1.48	−5.26	0.000	0.68	2.89	1.34	2.89	1.15	2.07	1.12
Female	2.21	0.71	3.32	0.47	3.87	1.29				2.91	1.33	2.93	1.16	2.2	1.05

**Table 5:** Comparison between vaccination for influenza and COVID-19 in percentages.

Gender	Men	Women
Flu vaccine	33.3%	26.7%
COVID-19 vaccine	85.5%	81.6%

**Table 6:** Comparison between vaccination for influenza and COVID-19 by age group in percentages.

Age	18–24	25–34	35–44	45–54	55–64	65 and Over
Flu vaccine	12.3%	19.2%	27.8%	25.0%	39.3%	75.6%
COVID-19 vaccine	76.2%	83.3%	72.2%	75%	94%	95.6%

**Table 7:** Correlations in skills, perceptions, attitudes and beliefs toward COVID-19.

	1	2	3	4	5	6
1. Mistrust of vaccine benefit	1	0.365 **	0.400 **	0.260 **	0.057	−0.195 **
2. Worries over unforeseen future effects	0.365 **	1	0.474 **	0.377 **	0.174 **	−0.157 **
3. Concerns about commercial profiteering	0.400 **	0.474 **	1	0.423 **	0.146 **	−0.208 **
4. Preference for natural immunity	0.260 **	0.377 **	0.423 **	1	0.148 **	−0.150 **
5. VL functional skills	0.057	0.174 **	0.146 **	0.148 **	1	−0.034
6. VL interactive/critical skills	−0.195 **	−0.157 **	−0.208 **	−0.150 **	−0.034	1

** The correlation is significant at the 0.01 (2-tailed) level.

**Table 8:** Regression model for Variable Mistrust of vaccine benefit.

Variables	β	b	SE of b	*t*	*p*
I believe vaccines are important in reducing or eliminating serious diseases	−0.315	−0.573	0.078	−7.35	0.000
Concerns about commercial profiteering	0.166	0.190	0.050	3.84	0.000
I believe more in natural immunity acquired through disease than in vaccines	0.134	0.166	0.052	3.20	0.001
I’m afraid of getting sick after getting vaccinated	0.124	0.154	0.051	3.04	0.003
VL interactive/critical skills	−0.093	−0.255	0.105	−2.42	0.016

β—standardized coefficient; SE—standard error; b—regression coefficient; *t*—*t*-test; R^2^—coefficient of determination. R^2^ adjusted = 0.31; F = 45.25; *p* < 0.001.

## Data Availability

The data presented in this study are available on request from the corresponding author. The data are not publicly available due to ethical reasons related to privacy, but the authors undertake to make the data available if requested for a valid reason.
